# Neurodevelopmental Underpinnings of Angelman Syndrome

**DOI:** 10.4172/1948-593X.1000111

**Published:** 2014-11-14

**Authors:** Guohui Li, Shenfeng Qiu

**Affiliations:** 1 Interdisciplinary Graduate Program in Neuroscience, School of Life Science, Arizona State University, USA; 2 Department of Basic Medical Sciences, University of Arizona College of Medicine-Phoenix, Phoenix, AZ 85004, USA

## Introduction

This review briefly discusses key recent research literature on Angelman Syndrome (AS), a rare genetic disorder of neurodevelopmental origin. Dysfunction/inactivation of the maternal *UBE3A* gene and its surrounding chromosome regions has been identified as the causative factor for AS. The human *UBE3A* gene is located within human chromosome 15q11-13 and encodes an E3 ubiquitin-protein ligase (UBE3A, also called E6 associated protein, E6-AP). Due to genetic imprinting of the paternal copy of *UBE3A* gene in many brain regions, loss of function of a single maternal copy of *UBE3A* is highly penetrant and pathogenic. Most of the deficits seen in AS patients have been reproduced in *Ube3a* gene maternal deficiency mice (‘AS mice’, *Ube3a*^m−/p+^), thus enabling mechanistic interrogations of AS pathogenesis and therapeutic explorations using mice models. Here we briefly discuss recent advances on AS etiology and identify some challenges in translating mechanistic insights into potential therapeutic interventions. Experimental evidence collected so far indicate impaired maternal *UBE3A* in neurons may contribute to AS deficiency by influencing multifaceted neural developmental processes including cell survival, synaptic transmission, signal transduction, gene expressions.

## Genetic Abnormality and Phenotypic Presentation

Angelman Syndrome (AS) is first reported and named thereafter by pediatrician Harry Angelman in 1965 [1]. AS is a severe debilitating neurodevelopmental disorder characterized by mental retardation, speech impairment, seizures, motor dysfunction, and a high prevalence of autism [2,3]. Bone abnormalities, such as brachycephaly, microcephaly, osteoporosis and delayed bone development-associated limb deformity and osteopenia are often co-occurring conditions [4–8]. AS influences the general population with an estimated rate of 1:10000 to 1:40000 in U.S. and the United Kingdom [9,10].

Loss of *UBE3A* gene function was identified as the cause of AS by two research groups in 1997 [11,12]. The human *UBE3A* gene encodes the E3 uniquitin ligase UBE3A. *UBE3A* gene is normally expressed in neurons only from the maternally inherited allele, while the paternal allele is silenced by epigenetic mechanisms known as imprinting. Therefore, mutation of the single maternal *UBE3A* allele in neurons leads to near complete loss-of-function of *UBE3A* gene. In the majority of AS patients, *UBE3A* gene is found inactivated by either intragenic mutation, chromosomal micro deletion in the 15q11-13 regions, paternal uniparental disomy (UPD), or a defective imprinting center (IC) that controls *UBE3A* transcription [13].

Consistent with this genetic architecture, targeted inactivation of *Ube3a* gene in mice [14] also support the role of *UBE3A* protein in AS; upon inheritance of the mutation through the maternal germline, *Ube3a* mutant mice (*Ube3a*^m−/p+^, ‘AS mice’) display salient pathological features of AS. Critical defects in both morphology and function of neurons was found [14]. It is important to note that while deficiency of *UBE3A* causes AS, increased *UBE3A* gene dosage (e.g. from maternal duplications of the *UBE3A*-spanning 15q11–q13 region [15] appears to be associated with intellectual and developmental abnormalities seen in autism spectrum disorders, and reproduce most autism features in mouse models [16,17]. However, it is currently not clear whether an increase in *UBE3A* dosage alone accounts for the autism phenotypes.

Molecular studies have revealed that knockdown of *Ube3a* in mouse increases neuronal death, which might be due to the accruement of p53 protein, p53-dependent transcription, or deposition of intracellular misfolded polyglutamine proteins [18]. In AS mouse models, the deficiency of *Ube3a* protein causes a reduction of dendrites spine density and dendritic length in multiple brain areas including hippocampus, cortex layer III-V and cerebellum [19,20] AS mice also show defects of dendrite polarization of pyramidal neurons in cortex and hippocampus, decreased dendritic arborization in cortex [21] and decreased synaptic vesicle density in hippocampus [22]. These morphological changes are consistent with the observed functional deficits. For example, decreased miniature excitatory postsynaptic currents (mEPSCs) and synaptic plasticity (such as longterm potentiation (LTP) impairment) are found in AS mouse models [23] which involves down-regulated N-methyl-D-aspartate receptor (NMDAR) function and deficiency of calcium influx. These evidence are indicative that UBE3A is required for normal neuronal activity. On the other hand, cellular UBE3A proteins levels are also affected by neuronal activity. Filonova et al. [24] recently reported that synaptic activation leads to dramatic changes *Ube3a* neuronal expression. Both increased neuronal activity by depolarization or fear conditioning behavioral paradigm enhanced neuronal *Ube3a* levels. The authors also found that in the absence of *Ube3a*, activity-dependent increase in ERK1/2 phosphorylation was impaired. It may be possible that this altered MAPK pathway may underlie the impaired synaptic plasticity and cognitive function in AS mice.

Intriguingly, experience-dependent maturation of excitatory cortical circuits, and visual cortex function associated with ocular dominance plasticity were found impaired in AS mice, suggesting *Ube3a* is necessary for maintaining developmental cortical plasticity and its loss-of-function may contribute to AS pathophysiology [20]. The same research group also reported that dysfunction of *Ube3a* resulted in deficits of fast-spiking inhibitory interneurons in cortex layer II-III and an abnormality of presynaptic vesicle release [25]. Consistent with the role of *Ube3a* in plasticity, a recent study demonstrated that the type 5 metabotropic glutamate (mGluR5) receptors-dependent LTD was potentiated in the hippocampus in AS mice [26]. It has been also reported that parvalbumin-positive (PV) interneurons in AS mice are more vulnerable than those of wild type mice in responding to chronic stress. Chronic stress treatment leads to more pronounced decrease of PV neurons in the hippocampus and basolateral amygdala of AS mice, a process that can be antagonized with fluoxetine [27]. AS mice also show abnormality in behavior related to the malfunction of basal ganglia circuits (e.g. instrumental conditioning). These mice have severe difficulty in initial acquisition of lever pressing, and were more habitual and impervious to changes compared with the wild-type control ones. Electrophysiological results revealed that both amplitude and frequency of mEPSCs are decreased in the dorsomedial striatum in AS mice, suggesting specific impairment of synaptic function in an associative corticostriatal circuit [28] that is also shared by the autism spectrum [29].

## Pathophysiological and Molecular Changes

A number of combined genetic and molecular studies have shed light on AS etiology. The UBE3A protein, first identified as the mediator of human papillomavirus types 16 and 18 E6 protein [30,31], regulates ubiquitin-mediated degradation of many proteins, such as the human homologues of yeast Rad23 (HHR23A), which is involved in DNA repair [32], the Src family member Blk [32] and the Rho-GEF pebble (pbl). Many other proteins are regulated by UBE3A when expressed in Drosophila [33]. These proteins include intracellular proteins such as misfolded polyglutamine proteins [18], annexin A1 [34] and the Hsp70/Hsc70 chaperones bound substrates [35] etc.

It has been found that various genetic mechanisms cause the loss (deletion or UPD), inactivation or mutations of maternal *UBE3A* gene (located in chromosome 15q11–13) [36], (Table 1). Imprinting for 15q11-q13 genes is controlled by a bipartite imprinting center (IC). This IC includes the Angelman syndrome imprinting center (AS-IC) and the Prader–Willi syndrome imprinting center (PWS-IC) [37–39]. Silencing the paternal copy of *UBE3A* gene is likely through paternal expression of a large antisense RNA transcript of *UBE3A* (*UBE3A*-ATS) and snoRNAs (small nucleolar RNAs) in neurons [11,12,40]. It was found that the two types of RNA transcript, sense and antisense, both the products of *Ube3a* gene, are expressed in a cell-type specific way in the brain. Neurons express maternal sense and paternal antisense, whereas glia express biallelical sense [41]. Furthermore, the disruption of maternal *Ube3a* gene resulted in an increase of paternal *Ube3a*-ATS in AS mouse model [42]. The *Ube3a*-ATS illustrated the inhibitory effect on the expression of paternal *UBE3A* gene [43], and is consistent with a large scale screening that revealed that maternal biased genes are significantly related to the developing brain [44]. Another study further support that the impaired function of *UBE3A* in AS patients is related to ubiquitin ligase instead of to its functional coactivator of transcription of the nuclear hormone receptor superfamily, such as the progesterone receptor (PR) [45]. The disturbance of the ubiquitin ligase activity gives rise to the impairment of protein ubiquitination [46]. A recent study showed that the expression level of paternal *Ube3a* is decreased in mouse neurons after the first postnatal week during which these neurons are undergoing rapid maturation. At the same time, the decrease of paternal *Ube3a* was accompanied by the nuclei accumulation of *Ube3a* of maternal origin. Interestingly, in contrast to neuron, glia cells (both astrocyte and oligodendrocyte) seem to express *Ube3a* biallelically [47].

The detailed mechanisms on how deficiency of *UBE3A* leading to AS are poorly understood. Studies using AS mice have provided some mechanistic insights by demonstrating that *Ube3a* plays a pivotal role in multiple CNS developmental processes, including cell cycle, signal transduction, transcription and synaptic plasticity [40]. One possible mechanism may be that changes of *Ube3a* expression can influence the viability of neurons. It was reported that the post mitotic neonatal neurons are decreased after maternal *Ube3a* inactivation in AS mouse hippocampus [48]. The loss of neurons may be due to either impaired metabolism or the disturbance of genes involved in cell death process [49], or both. It was also shown that mitochondria in AS mouse exhibited a smaller size in the hippocampus and a partial oxidative phosphorylation defect in the whole brain [22]. Another study revealed that proliferation of neurons was disrupted in AS mice due to the increased expression of cyclin-dependent kinase inhibitor p27, whose degradation is mediated by the *Ube3a* [50].

Another potential mechanism is that protein synthesis including receptors expression can be affected by *Ube3a* dysfunction. A recent study showed that the Golgi apparatus (GA) cistern was swollen and disorganized in *Ube3a* maternal deficiency mice, and the pH in GA lumen is increased in cortical neurons. This has implication that a less acidified GA would result in impaired protein sialylation and secretion mechanisms [51]. Previous researches also showed that *Ube3a* regulate the degradation and turnover of RhoA-GEF Ephexin-5, activity-regulated cytoskeleton-associated protein (Arc), p53, and p27 via ubiquitination [13,52,53]. It has been reported that Arc protein can promote endocytosis of the α-amino-3-hydroxy-5-methyl-4-isoxazolepropionic acid-type glutamate receptor (AMPAR), thereby reducing cell surface functional glutamate receptors by facilitating their interaction with dynamin and endophilin [54]. It is not surprising that AMPAR quantity at excitatory synapses was also found decreased, in correlation with an increase of Arc expression after *Ube3a* function was disturbed in neurons [20,55].

Another hypothetic cause is that *Ube3a* abnormality disturbs the regulation of gene expression. A recent study demonstrated that levels of both Ring1B, which ubiquinates nucleosomal histone H2A to regulate gene expression, and histone H2A, are elevated in many tissues in *Ube3a* knockout mice [56]. One recent study found that 7 genes are increased and 57 genes are decreased in AS mouse. These genes are functioning in signal transduction, nervous system development and cell death. Some of those genes *(Fgf7, Glra1, Mc1r, Nr4a2, Slc5a7 and Epha6)* are confirmed of relevant with AS phenotype [49]. It was also shown that elevated Arc level in AS mouse disturbs the brain-derived neurotrophic factor (BDNF) to recruit the postsynaptic density-95 (PSD-95) protein, disrupts association of PSD-95 with TrkB, and the association of PLCγ and Grb2-associated binder 1 (Gab1) with TrkB, therefore impairing BDNF, TrkB and PI3K-Akt pathways [57]. Another recent study found that the expression of α1 subunit of sodium/potassium-ATPase (α1-NaKA) is increased in hippocampus in AS mouse. The abnormal expression likely explains a series of changes such as elevated axon initial segment proteins and altered membrane properties including resting potential, threshold potential and action potential. These alterations were corrected by reducing α1-NaKA genetically [58]. This study suggests loss of *Ube3a* leads to changes in neuronal excitability likely through altered membrane biophysical properties.

## Therapeutic Explorations

The current efforts in therapeutic exploration for AS have been taken on identified putative pathological basis. A conspicuous idea would be to restore the function of the *UBE3A* in the brain, by retrieval either maternal or paternal copy of the gene. For restoration of maternal *Ube3a*, one study used recombinant adeno-associated virus (rAAV) to introduce type 2 terminal repeat (TR2) flanked *Ube3a* into the hippocampus of adult AS mice. The study found that rAAV restored the level of *Ube3a* in AS hippocampus, rescued the impaired of LTP, and enhanced the cognitive learning as evaluated by Morris Water Maze test [59]. These rescue experiments suggest that neuronal circuit deficits can arise from lack of *Ube3a* function per se, and restoration of *Ube3a* expression could potentially overcome certain aspects of developmental deficits.

The existence of the intact paternal *UBE3A* allele has the intriguing implication that activation of the silent allele may be able to fulfill the functions of the missing maternal ones, in a way analogous to rescuing neural deficits in adult *Mecp2* knockout mice by reinstating the functional *Mecp2* gene [60]. In an elegant genetic study, Meng et al. showed that inhibition of *Ube3a*-ATS expression both *in vivo* and *in vitro* could elevate expression of paternal *Ube3a* [61]. The activation of paternal *Ube3a* could be achieved by blocking the paternal *Ube3a*-ATS with poly-adenine cassette insertion in AS mouse models. Many resulting AS deficiencies, such as impaired LTP, cognitive deficits, and motor dysfunction were ameliorated [61]. Restoring the paternal *Ube3a* expression through non-genetic approaches also seems to hold great promises. Through chemical library screening, Huang et al. (2012) have found that several topoisomerase inhibitors, such as topotecan and irinotecan could resuscitate paternal *Ube3a* and rescue cellular function in neurons [62]. Although topoisomerase inhibitors lack specificity on neurons and are likely to be toxic to many tissue types, this study represents a major conceptual breakthrough by showing that rescuing the dysfunctional *UBE3A* gene in brain can be achieved through bypassing the genetic manipulations.

Maternal *Ube3a* deficiency in mice is known to impair synaptic transmission and interfere with a critical molecular player in synaptic plasticity, Ca^2+^/calmodulin-dependent protein kinase II (CaMKII) [20,23]. Weeber et al. showed that AS mice had impaired hippocampal long-term potentiation (LTP) and reduced context-dependent learning, which is correlated with an increased CaMKII phosphorylation at Thr305/Thr306 inhibitory sites and a reduced kinase activity [23]. In a following study [63], the same group further crossed female AS mice with heterozygous males that carried the targeted CaMKII-T305V/T306A mutation, a genetic manipulation that prevents inhibitory phosphorylation of CaMKII and elevates CaMKII activity. Intriguingly, a reduction of CaMKII inhibitory phosphorylation was able to rescue the motor deficits, seizures, LTP impairment and the hippocampus dependent learning. Collectively, these findings indicate misregulation of CaMKII may be a molecular substrate underlying the neurobehavioral deficits in AS. The notion that restoring affected signal transduction pathways may alleviate AS pathology is also supported by another recent study [57]. Cao et al. reported that altered LTP in AS mouse model can be corrected after the TrkB signal pathway was restored by using a bridged cyclic peptide (CN2097) to interfere the interaction between the increased Arc and PSD-95.

## Outstanding Questions and Major Challenges

Despite these emerging successes in restore neural functions in AS mouse models, outstanding questions and challenges remain in the field. For example,

1. What is the definite role of UBE3A in neural connections or circuits within and between many brain regions, and in what molecular context is UBE3A involved to regulate synaptic development, transmission, and plasticity? Why increased *UBE3A* dosage is more represented in autism spectrum disorders [16,17] ?
2. How does UBE3A differentially affect both excitatory and inhibitory synapses, favoring an enhanced local circuit hyper-excitability [20,25]?
3. The molecular mechanisms by which *UBE3A* deficiency lead to AS remain enigmatic. The protein substrates of UBE3A in neurons remain to be identified [10]. Arc, Sacsin, HHR23A and Ephexin 5 represent only a small number of proteins known to be directly regulated by UBE3A in *neurons*. Revealing more neuronal molecular substrates or interactomes and how deficiency of maternal *UBE3A* disrupts cellular homeostasis can be illuminating for AS pathogenesis and molecular intervations.
4. The dramatic variations of symptoms among AS patients imply the contributions of other elusive and perhaps much more complex causes other than maternal *UBE3A* dysfunction. For example, other genes such as GABA_A_ receptor β3 subunit (*GABRB3*) gene which locates within the chromosome 15q11-13 locus have been proved playing a role in the AS development. The impaired expression of *GABRB3* can render featured phenotypes of AS in mice. These results raised the questions of the definite role of the *GABRB3* and its relation to *UBE3A* in AS genesis [64].
5. Some discrepancies exist between AS model mouse behavior and AS patient clinical features. AS mice showed normal social seeking and activity level in contrast to the frequently observed behavioral deficits of AS patients [65]. This may be explained by the larger size of genetic defect in patients than that of the AS mice. Further observations on the variations in eating behavior and body growth among patients with different genetic deficits, specifically patients with big deletion or ones with UPD implied that other factors within the 15q11-13 locus may play a role in the pathogenesis too [36,66].
6. On the forefront of AS therapeutic endeavors, the potential of topoisomerase inhibitors in restoring *UBE3A* expression and correcting AS pathophysiology awaits further experimental validation and extrapolation.

## Figures and Tables

**Table 1:** Ascertained genetic abnormalities in AS*.

Genetic abnormality	Percentage in AS cases
Maternal deletion of 15q11-13 (*De novo*)	~70%
Paternal UPD	2–5%
Imprinting defects	2–5%
Mutations/variants of *UBE3A* gene	~5–10 %
Other causes unidentified	~10 %

**Note:** According to a report by Ramsden et al. 2010, and also based on the data from the public database Decipher (https://decipher.sanger.ac.uk).
